# Trans-kingdom conservation of mechanism between bacterial actifensin and eukaryotic defensins

**DOI:** 10.1038/s44259-025-00135-x

**Published:** 2025-07-22

**Authors:** Ivan Sugrue, Carolin Ade, Paula M. O’Connor, Jan-Martin Daniel, Paolo Innocenti, Nico Kirsch, Nathaniel I. Martin, Günther Weindl, Colin Hill, Tanja Schneider, R. Paul Ross

**Affiliations:** 1 APC Microbiome Ireland, University College Cork, Cork, Ireland; 2 School of Microbiology, University College Cork, Cork, Ireland; 3 Institute for Pharmaceutical Microbiology, University of Bonn, University Hospital Bonn, Bonn, Germany; 4 Teagasc Food Research Centre, Moorepark, Fermoy, Co. Cork, Ireland; 5 German Center for Infection Research (DZIF), Partner Site Bonn-Cologne, Bonn, Germany; 6 Biological Chemistry Group, Institute of Biology, Leiden University, Leiden, The Netherlands; 7 Department of Pharmacology and Toxicology, Pharmaceutical Institute, University of Bonn, Bonn, Germany

**Keywords:** Microbiology, Antimicrobials, Bacteria, Peptides

## Abstract

Antimicrobial peptides are defense molecules found across all domains of life holding promise for developing therapies against drug-resistant pathogens. Actifensin, from *Actinomyces ruminicola* DPC7226, exhibits potent activity against gram-positive bacteria and shares structural similarities with eukaryotic defensins. This study characterized actifensin’s mechanism of action and therapeutic potential. The findings revealed that actifensin inhibits peptidoglycan synthesis by binding lipid II (*Kd* = 30 ± 20 nM). Unlike defensins, it also binds lipid I (*Kd* = 24 ± 27 nM) without significant difference, suggesting the N-acetyl glucosamine moiety of lipid II is not required for complexation. Membrane disruption was not observed with DiSC_3_(5) fluorescence, or synthetic unilamellar liposomes, indicating indirect cell death via cell wall weakening, visualised by phase contrast microscopy. Actifensin showed no haemolytic activity or toxicity up to 128 µg/ml in human erythrocytes and Hep G2 cells. The peptide was not immunogenic, demonstrating no induction of LDH release in PBMCs or any effect on TLR-mediated signalling. Structural motif analysis identified actifensin as part of a conserved trans-kingdom defensin subfamily, GXGCP, distinct from XTCD peptides in more recently evolved arthropods. These findings emphasise the conserved structure-function relationship of antimicrobials across kingdoms, suggesting a shared evolutionary history of defensins and highlight the therapeutic potential for them or their variants.

## Introduction

Antimicrobial peptides are a ubiquitous form of host defence between and against microbes, and are produced across the super-kingdoms of life, eukarya, prokarya, and archaea. In light of the antimicrobial resistance crisis, antimicrobial peptides represent a reservoir of tools which could be developed into additional novel antimicrobial therapies. In prokaryotes, genome-encoded and ribosomally-produced antimicrobial peptides of bacteria, bacteriocins, are a heterogeneous group of peptide superfamilies displaying a range of structures from simple to complex, with diverse mechanisms and applications^1^. Eukaryotes produce defensins, structurally-related peptides of the innate immune system, represented by multiple protein families consisting of the α-, β-, and θ-defensins of mammals, plant defensins, and the cysteine-stabilised αβ defensins, known as invertebrate or arthropod defensins, from fungi and invertebrates^2^.

The widespread and conserved nature of tertiary defensin structures, consisting of variations on an antiparallel β-sheet against an α-helix stapled with disulphide bonds, implies a shared evolutionary heritage, and their origin is considered to have been convergent for at least two major superfamilies^2^. The cysteine-stabilised αβ (CSαβ) defensins, consisting structurally of an alpha-helix connected to a β-sheet via three disulphide bonds, are a family of anti-gram positive peptides produced by distantly related phyla of ascomycete fungi^3^, ancient invertebrates such as molluscs^4^, and arthropods such as flies^5^, which have evolved over hundreds of millions of years. The specific mechanisms of action of several defensins have been elucidated, such as fungal plectasin which is known to bind to the bacterial cell wall precursor molecule, lipid II, preventing incorporation into the growing peptidoglycan structure, arresting cell wall biosynthesis and eventually leading to cell death^3^. More recently, it has been suggested that it forms Ca^2+^-sensitive supramolecular assemblies at the cell membrane following lipid II binding, leading to membrane depolarisation and consequent death^6^. Other defensins have also been shown to act via peptide interaction with lipid II, including human β-defensin 3^7^ and the fungal defensin eurocin^8^.

As a heterogeneous group of peptide superfamilies, bacteriocins are far more diverse in structure and display diverse mechanisms of action, many of which remain to be fully characterised. Nisin, the most well-studied bacteriocin, has a dual mechanism of action, first binding lipid II and subsequently forming pores in cell membranes^9^. Other bacteriocins interact with exposed cell surface proteins such as essential sugar or protein transporters, as is the case for pediocin PA-1 with the mannose transporter in gram-positives^10^ or darobactin A which inhibits the activity of BamA in gram-negatives^11^.

Recently we described actifensin, a member of the first family of CSαβ defensin-like bacteriocins encoded within the genomes of, and produced by, the genus *Actinomyces* of the bacterial phylum Actinomycetota, formerly known as Actinobacteria^12^. Concurrently with the description of actifensin, 47 homologous peptides were identified encoded within the genomes of related species, sharing as little as 52% mean amino acid identity, and representing an arsenal of potential new antimicrobials. Actifensin displays potent activity against gram-positive pathogenic bacteria with potential for therapeutic development.

With the necessity for novel antimicrobial therapies, we sought to characterise the specific target of the CSαβ-defensin-like bacteriocin actifensin. Here, we investigate the mechanism of action of actifensin, identifying the cell wall as a target, and further show interaction of the peptide with the peptidoglycan precursor, lipid II, like CSαβ defensins. We demonstrate therapeutic qualities of actifensin through lack of cytotoxicity and red blood cell lysis and lack of resistance development. In parallel, we explored the conservation of CSαβ defensin features across invertebrate phyla and bacteria, split across two distinct subfamilies, indicating a trans-kingdom conservation of mechanism and (secondary) structure between CSαβ defensins, including actifensins.

## Results

### Actifensin directly complexes with the peptidoglycan precursor molecule lipid II

**Fig. 1:**
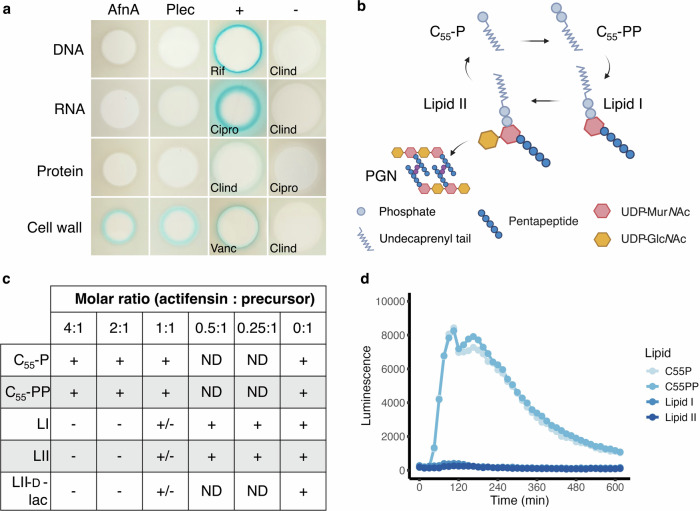
Actifensin complexes with the peptidoglycan precursor molecule, lipid II. **a** Spot-on-lawn assays using stress-response bioreporter strains of *Bacillus subtilis* 168: pAC6-P_yorB_ (DNA), pAC6-P_yvgS_ (RNA), pAC6-P_yheI_ (protein), and pAC6-P_ypuA_ (cell wall). Actifensin (AfnA) and plectasin (Plec) induce β-galactosidase expression from the *ypuA* promoter, indicating interference with cell wall biosynthesis. Results are compared with control antibiotics: Rif (rifampicin), Clind (clindamycin), Cipro (ciprofloxacin), and Vanc (vancomycin). **b** Basic schematic of cell wall precursor compound structures and the cycle of their incorporation into peptidoglycan. PGN peptidoglycan. **c** Results of lipid complexation assays using purified cell wall precursors at varying molar ratios of actifensin to lipid. Complexation was assessed based on the lipid band intensity on the membrane: absence (−), decreased intensity (+/−), or unaffected intensity (+). ND not determined. **d** Antagonization assays of the actifensin-induced *lia* cell wall stress response in *B. subtilis* P_lia_-lux, performed at 1:1 molar ratios of cell wall precursor lipids to actifensin.

Initially we sought to determine the mechanism of action of actifensin using *Bacillus subtilis* reporter strains, which are sensitive to the bacteriocin. Purified actifensin was spotted onto Mueller Hinton agar seeded with the *B. subtilis* strains expressing β-galactosidase under control of the promoters for proteins induced by selective interference with major biosynthesis pathways^13^ that have been used previously to identify broad targets of antimicrobial activity^14,15^.Five micrograms of pure actifensin produced zones of inhibition against all four strains and induced formation of a blue halo of the strain *B. subtilis* 168 pAC6-P*ypuA*, indicating specific induction of the cell-wall stress response and the cell wall as a putative target for actifensin action (Fig. 1a). *B. subtilis* expressing β-galactosidase under control of the *yorB, yvgS*, *yheI* promoters (DNA, RNA, and protein stress response pathways, respectively) produced no blue colour, indicating lack of induction of those stress responses.

Given the similarity in sequence and predicted structure of actifensin to known lipid II-binding peptides, as well as bioreporter results suggesting interaction with the cell wall, we investigated whether actifensin complexes with precursor lipids involved in cell wall biosynthesis using thin-layer chromatography (Fig. 1b, c). Actifensin specifically complexed with the precursors lipid I and lipid II at 1:1 molar ratios (Fig. 1c and Supplementary Fig. 1). The lipid II variant, lipid II-d-lac, also complexed with actifensin at a 1:1 ratio, visible as a lower intensity band indicating binding with reduced affinity, which was absent with a 2:1 ratio of actifensin:lipid (Fig. 1c and Supplementary Fig. 1). No complex formation was observed with the lipid carriers, C_55_-P and C_55_-PP, in the presence of actifensin at any ratio, indicating lack of affinity of the peptide for the undecorated lipids (Fig. 1c and Supplementary Fig. 1).

Actifensin was tested against a *B. subtilis* P_*lia*_*-lux* bioreporter strain known to be induced in response to **l**ipid II-**i**nterfering **a**ntibiotics (Lia), such as vancomycin^16^. Actifensin treatment produced a strong luminescent response at all concentrations tested (0.125–16 µg/ml) indicative of LiaRS stress response induction, pointing to interference with the lipid II biosynthesis pathway (Supplementary Fig. 2). The response was concentration-dependent from 0.125–8 µg/ml as was observed for vancomycin at all concentrations (Supplementary Fig. 2). The presence of lipid II at a 1:1 molar ratio (lipid:peptide) was sufficient to fully antagonise the luminescent response, further corroborating direct peptide-lipid II complexation and lipid II as a target for actifensin (Fig. 1d and Supplementary Fig. 3). The response was also eliminated in the presence of lipid I at a 1:1 ratio suggesting the UDP-Glc*N*Ac (*N-*acetyl glucosamine) sugar moiety which is present in lipid II is not essential for stable complexation as has been suggested for the defensin plectasin^3^. The undecorated lipid carriers C_55_-P and C_55_-PP did not affect the response, indicating lack of interaction with actifensin, corroborating TLC data and further ruling them out as targets for the peptide (Fig. 1d). Isothermal titration calorimetry showed that actifensin binds lipids I and II with high affinity (Kd = 24 ± 27 and 31 ± 20 nM, respectively) (Supplementary Fig. 4).

### Treatment with actifensin weakens the bacterial cell wall without direct membrane interaction

**Fig. 2:**
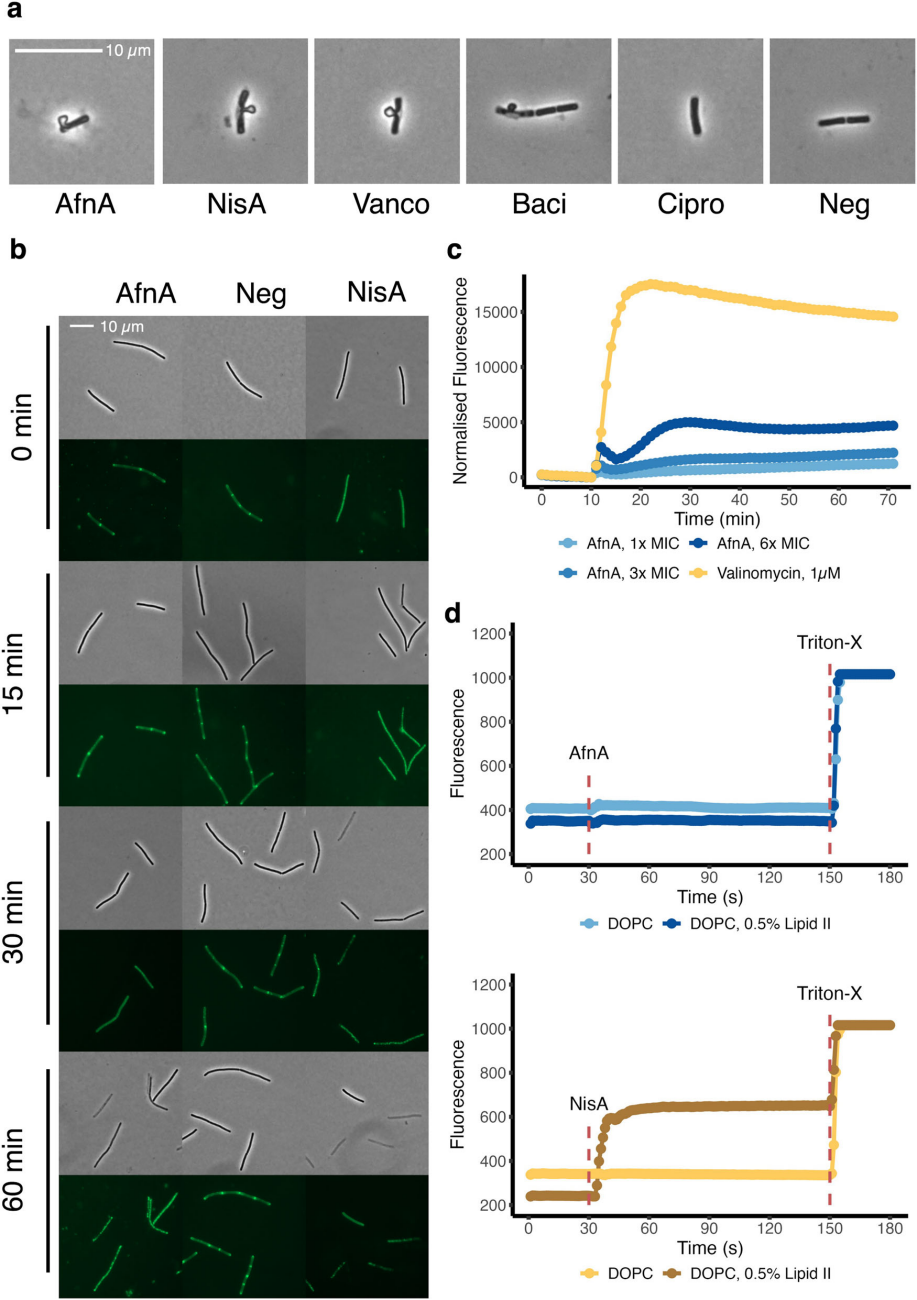
Actifensin’s action interferes with cell wall biosynthesis without directly impacting the cell membrane. **a** Phase contrast microscopy images of *B. subtilis* 168 treated with mechanistically distinct compounds. Lipid II-binding agents induce a weakened cell wall resulting in membrane blebs. **b** Phase contrast images of *B. subtilis* P_*xyl*_*-gfp-minD* over 60 min following treatment with actifensin, the known membrane-interacting bacteriocin, nisin, and no treatment. **c** DiSC_3_(5) fluorescence of *B*. subtilis 168 cells treated with increasing concentrations of actifensin and the membrane-depolarising compound valinomycin. **d** Carboxyfluorescein (CF) efflux assays from unilamellar liposomes lack of CF release with actifensin in the presence and absence of lipid II, where nisin forms pores in the presence of lipid II.

Treatment of *B. subtilis* with actifensin at 2 µg/ml produced cell deformations visible as membrane blebs (Fig. 2a), indicative of a detrimental effect on cell wall integrity that is characteristic of peptidoglycan biosynthesis inhibiting antibiotics^3,14^. Membrane blebs were also produced following treatment with known cell-wall targeting compounds nisin, vancomycin, and bacitracin, but not with the ciprofloxacin control (Fig. 2a).

We sought to establish if actifensin also targeted the cell membrane directly in addition to interfering with cell wall biosynthesis, a feature of some other amphipathic lipid II binding peptides such as nisin^17^. The effects of actifensin and nisin treatments on *B. subtilis* expressing GFP-tagged MinD were visualised over the course of 60 min (Fig. 2b). Actifensin caused delocalisation of the MinD protein from the poles following 30 min of treatment with 12.5 µg/ml, indicating membrane depolarisation. The cell division protein MinD is known to bind to the cell membrane at the poles of the cell, requiring membrane potential for cellular localisation^18^. As such depolarisation, visualised as a delocalisation of GFP foci in the cell can be interpreted as destruction of membrane potential through pore formation and/or cell death. In contrast with the delayed effect observed with actifensin, treatment with nisin, the known pore-forming peptide rapidly delocalised MinD, and the untreated control displayed localised MinD throughout. Membrane potential was largely unaffected by actifensin when measured by fluorescence of DISC_3_(5) dye release over the course of 1 h (Fig. 2c). The hydrophobic voltage-sensitive dye accumulates in energised cells, quenching fluorescence, which is rapidly released upon membrane depolarisation^19^, as was seen with the depolarising antibiotic valinomycin (Fig. 2c). Lack of membrane depolarisation by actifensin was further confirmed by assaying carboxyfluorescein release from synthetic unilamellar liposomes formulated with lipid II (Fig. 2d). Treatment with actifensin did not induce a release of carboxyfluorescein, whereas nisin induced a rapid release of the dye following addition (Fig. 2d). The results from the DISC_3_(5) assay and artificial membrane (liposome) experiments confirm that membranes are not the target of actifensin, and MinD delocalisation after 30 min of treatment results from a compromised cell wall as a consequence of actifensin-mediated biosynthesis inhibition.

### Actifensin displays low toxicity and immunomodulatory capacity towards human cells

Concurrently with these mechanistic studies, we investigated characteristics of actifensin’s applicability as a therapeutic. First, we sought to establish actifensin activity against a panel of gram-positive and gram-negative pathogens and found that actifensin inhibited gram-positive pathogens in the range of 0.06 µM (*Micrococcus luteus*) to 1.95 µM for clinically-relevant vancomycin-intermediate *Staphylococcus aureus* (VISA), and vancomycin-resistant enterococci (VRE) (Table 1). In addition, we tested the MIC of actifensin in the presence of calcium cations, as the defensin plectasin has been described to form supramolecular assemblies at the cell membrane in the presence of Ca^2+^, increasing killing activity^6^. The MIC of actifensin remained unchanged when 1.25 mM Ca²⁺, equivalent to physiological serum concentrations, was added for six *Staphylococcus* strains and *M. luteus* (Supplementary Table 1). Actifensin also inhibited formation of *S. aureus* ATCC25923 biofilms at twofold the MIC value (0.98 µM) (Supplementary Fig. 5).

**Table 1:** Minimum inhibitory concentrations (MICs) of actifensin and plectasin

Species	Strain	Description	Actifensin MIC	Plectasin MIC	Fold difference^a^
			µg/ml (µM)	µg/ml (µM)	
*Bacillus subtilis*	168	WT strain	2.0 (0.49)	0.25 (0.06)	0.125x
*Enterococcus faecium*	APC1031	WT strain, VRE (vanB)	4.0 (0.98)	128 (31.7)	32x
*Escherichia coli*	O-19592	WT strain	>64 (>15.6)	>64 (>15.8)	–
*Escherichia coli*	∆tolC	Mutated efflux protein	>64 (>15.6)	>64 (>15.8)	–
*Escherichia coli*	∆wecA	LPS synthesis mutant	>64 (>15.6)	>64 (>15.8)	–
*Micrococcus luteus*	ATCC4698	WT strain	0.25 (0.06)	ND	–
*Moraxella catarrhalis*	ATCC43617	WT strain	16 (3.91)	ND	–
*Staphylococcus aureus*	HG001	WT strain	4.0 (0.98)	64 (15.8)	16x
*Staphylococcus aureus*	HG001 DapR	Daptomycin resistant	4.0 (0.98)	16 (3.98)	4x
*Staphylococcus aureus*	SA113	WT strain	4.0 (0.98)	8.0 (1.98)	2x
*Staphylococcus aureus*	SA113 ∆tarO	WTA synthesis mutant	4.0 (0.98)	8.0 (1.98)	2x
*Staphylococcus aureus*	SA113 ∆LTA	LTA mutant	4.0 (0.98)	8.0 (1.98)	2x
*Staphylococcus aureus*	SA113 ∆dlt	TA synthesis mutant	1.0 (0.24)	0.25 (0.06)	0.25x
*Staphylococcus aureus*	RN4220	WT strain	4.0 (0.98)	16 (3.98)	4x
*Staphylococcus aureus*	RN4220 ∆tarS/M	WTA synthesis mutant	4.0 (0.98)	32 (7.92)	8x
*Staphylococcus aureus*	Mu50	MRSA/VISA	8.0 (1.95)	>64 (>15.8)	>8x
*Staphylococcus aureus*	137/934	VISA	8.0 (1.95)	128 (31.7)	16x
*Staphylococcus aureus*	VC40	VISA	4.0 (0.98)	128 (31.7)	32x
*Staphylococcus aureus*	SG511	Hypersusceptible strain^b^	1.0 (0.24)	0.25 (0.06)	0.25x
*Staphylococcus simulans*	22	WT strain	0.25 (0.06)	8.0 (1.98)	32x
*Streptococcus pneumoniae*	R6 (ATCC BAA-255)	WT strain, avirulent	0.125 (0.03)	1.0 (0.25)	8x

*ND* not determined.

^a^Fold difference in activity of actifensin compared to plectasin.

^b^Multiple mutations in regulatory genes^3^.

**Fig. 3:**
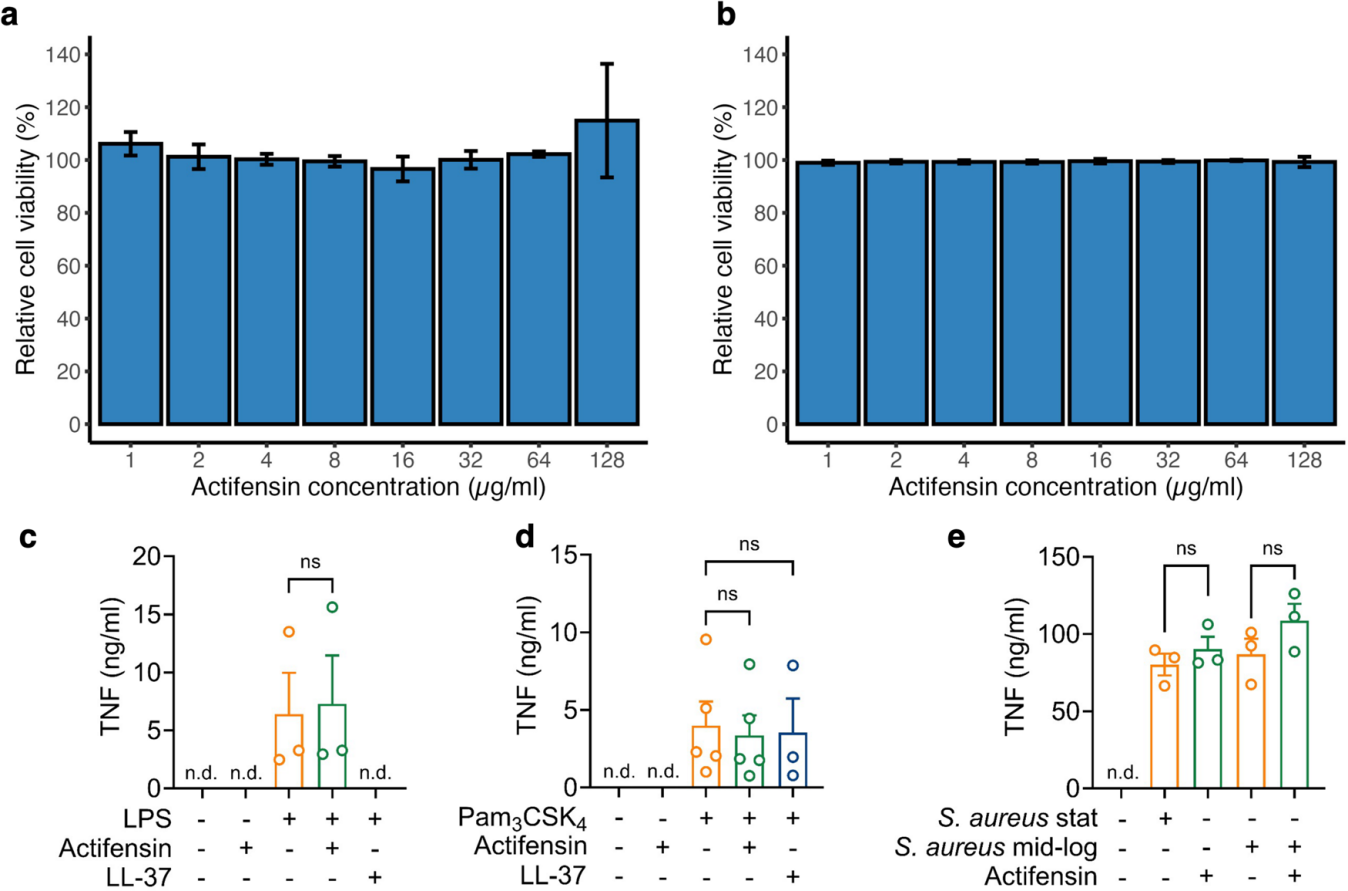
Actifensin’s effects on human cells. **a** Relative viability of human epithelial cells (Hep G2) following 24 h of actifensin treatment, calculated as a percentage of untreated cells. **b** Relative viability of human erythrocytes following actifensin treatment for 24 h. Actifensin does not modulate TNF release in PBMCs stimulated with **c** LPS, **d** Pam3CSK4, or **e** heat-inactivated *S. aureus* (6.5 × 10^8^ cfu/ml in stationary phase or 6.9 × 10^8^ cfu/ml in mid-logarithmic phase). Bar graphs show mean + SEM (*n* = 3–5). n.d. not detected.

To further evaluate the therapeutic potential of actifensin, we assessed its effects on human cells in vitro. Actifensin showed no haemolytic activity against erythrocytes and no toxicity toward human epithelial Hep G2 cells after 24 h of treatment at concentrations up to 128 µg/ml (31.3 mM) (Fig. 3a, b). Cationic antimicrobial peptides, including defensin-like peptides, are also known to exhibit immunomodulatory properties in the absence of cytotoxic effects by interacting with bacterial pathogen-associated molecular patterns (PAMPs) such as lipopolysaccharides (LPS) and lipoproteins, altering cellular signalling^20^. Therefore, we aimed to investigate the role of actifensin in modulating interactions with host immune cells. In peripheral blood mononuclear cells (PBMCs), actifensin did not induce lactate dehydrogenase (LDH) release at concentrations up to 20 µM (Supplementary Fig. 6) and further indicating a lack of toxicity towards mammalian cells. To study Toll-like receptor (TLR) 4- and TLR2-mediated signalling, we used specific ligands: LPS for TLR4 and Pam_3_CSK_4_ for TLR2. The human cathelicidin LL-37, known for its ability to neutralise endotoxins, was used as a control to inhibit TLR4 downstream signalling^21^. As expected, LL-37 inhibited LPS-induced TNF secretion but did not affect Pam_3_CSK_4_-induced TNF secretion, whereas actifensin showed no effect on either (Fig. 3c, d). To better replicate the complexity of the bacterial cell wall, we used heat-inactivated *Staphylococcus aureus* (*S. aureus*) cells. Cell wall teichoic acid (WTA), a key cell wall polymer of *S. aureus*, plays an important role in infection and inflammation^22^, with its production varying by bacterial growth phase^23^. To evaluate actifensin’s effect on WTA-induced TNF secretion, heat-inactivated *S. aureus* cells from both stationary and mid-logarithmic growth phases were tested. Cells from both phases similarly induced TNF secretion from PBMCs, which remained unaffected by actifensin (Fig. 3e).

### Actifensins comprise part of a trans-kingdom structural subfamily of the arthropod defensins

**Fig. 4:**
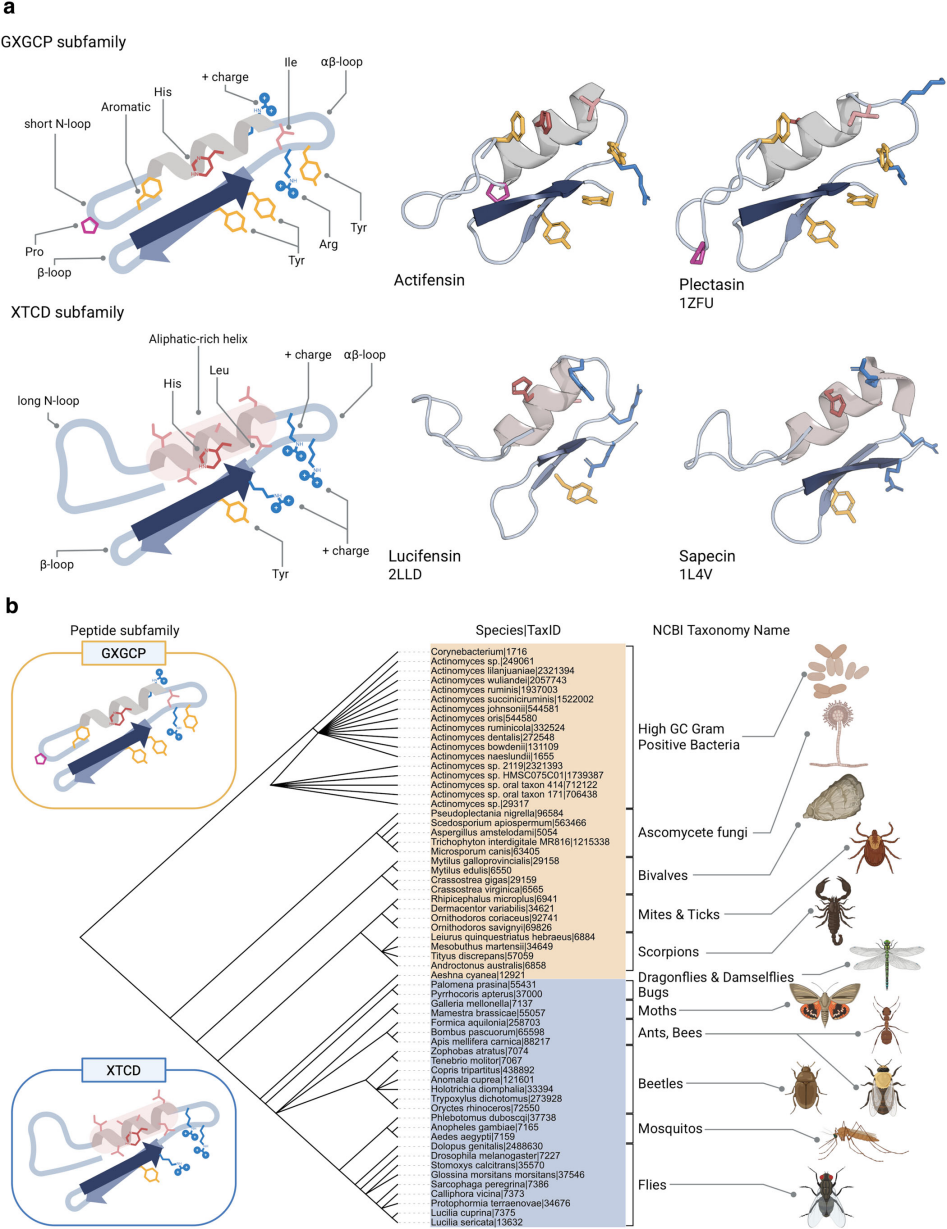
Conserved structures and spread of the GXGCP and XTCD subfamilies of the CSαβ peptides. **a** Schematic and exemplary peptide structures displaying conserved features and mechanism-related residues separating the peptide subfamilies. **b** Taxonomic phylogram of species that encode CSαβ peptides coloured by the type of peptides produced (yellow—GXGCP, blue—XTCD) and images displaying spread of peptide presence across phyla.

Given the conserved nature of actifensins and related eukaryotic CSαβ defensins, we investigated the conserved structures of peptides with known lipid II binding activity. To do so, protein sequences of the arthropod defensin protein family, spanning diverse eukaryotic phyla, were acquired and aligned to actifensin and its bacterial homologs. The peptide sequences separated into two groups based on conserved motifs in the N-terminal loop and alpha helical regions of the peptides (Fig. 4a). The peptide sequences ranged from 34 to 60 residues (mean 38) but 119 of the 123 peptides measured 43 residues or less (Supplementary Fig. 7). Eighty-eight peptides (71.5%), including the actifensins and eukaryotic peptides of diverse phyla, present a shorter N-terminal loop of which 52 have an N-terminal motif beginning with GXG, where X is an aromatic residue, present in actifensin as Gly1-Phe2-Gly3, followed by the first disulphide-forming cysteine residue at position 4. In 80/88 of these peptides, hereafter referred to as the GXGCP group, the proline is present at position 5. Of the remaining 35 peptides (non-GXGCP), 33 grouped with a conserved N-terminal motif of XTCD and two aliphatic residues, such as Leu-Leu in 23/33 peptides. These peptides, hereafter referred to as the XTCD group, are present only in eukaryotes and feature an extended N-terminal loop relative to the GXGCP subfamily, that lacks the conserved proline residue and aromatic residue at position 2. In addition, the XTCD group contain an aliphatic-rich helix with particular propensity for a double-Ala motif between cysteines 2 and 3. This contrasts with the less conserved helix-forming region of the GFGCP group. Across both subfamilies, a single conserved histidine (72/88 peptides) precedes the third disulphide forming cysteine within the alpha helix (Fig. 4 and Supplementary Fig. 7).

Several residues in plectasin have been linked mechanistically with its lipid II binding action including F2, H18, K20, I22, K23, Y25, K26, Y29, and Y40 in distinct binding models^3,6^. Each of these residues, except K23, is largely conserved or subject to conservative substitution in actifensin and other GXGCP peptides implying lipid II-binding conservation of function across peptides (Fig. 4a). In the XTCD group some of these key residues are subject to non-conservative substitutions, such as absence of an aromatic side-chain in the N-terminal loop (F2 in actifensin and plectasin) and the substitution of aromatic residues Y25 and Y40 with positively-charged arginine and lysines.

Plotting the spread of the GXGCP and XTCD peptide subfamilies on the tree of life reveals a separation of the peptides across phylogenetic history (Fig. 4b). The GXGCP group are a trans-kingdom subfamily present in bacteria and ancient invertebrate eukaryotes such as Ascomycete fungi molluscs, and primitive insects dragonflies and scorpions. Defensins within these eukaryotic phyla have previously been described as ancient invertebrate-type defensins (AITDs)^24^. The XTCD group, some of which have been previously described as classical insect type defensins (CITDs) are restricted to more recent phylogenetic orders including Coleoptera (beetles), Diptera (flies), Hemiptera (true bugs), Hymenoptera (wasps, bees, ants) and Lepidoptera (butterflies, moths) (Fig. 4b).

## Discussion

This study provides insights into the mechanism of action of actifensin and its potential as a therapeutic agent. Through complementary approaches, we describe actifensin’s ability to directly complex with the peptidoglycan precursor lipid II, inhibiting bacterial cell wall biosynthesis in a mechanism of action that is similar to other known lipid II-binding peptides. As a key structural element of the bacterial cell wall lipid II remains a therapeutic target for drug design and delivery^25^, and several new antimicrobial compounds have been identified and characterised in recent years targeting various peptidoglycan precursor compounds via distinct mechanisms. In the case of actifensin, we found complexation between both lipid I, and lipid II, but not C_55_P or C_55_PP, indicating a requirement for the *N*-acetyl muramic acid sugar with the pentapeptide sidechain for binding. Defensins and homologous bacterial*-*derived peptides, have been shown to bind components of lipid II such as the UDP-MurNAc-pentapeptide^26^. In the case of plectasin, the second sugar of lipid II (UDP-GlcNAc) stabilises the lipid-peptide complex^3^. However, ITC data show that for actifensin the presence of the second sugar does not improve binding or stability of the lipid-actifensin complex (Supplementary Table 2 and Supplementary Fig. 4) which may be related to the structural differences in the peptides such as the lack of a negatively charged patch or residues that are present in other peptides such as plectasin.

Actifensin was also able to complex with the lipid-II variant lipid-II-d-lac, in which the d-Ala-d-Ala terminus of the pentapeptide is replaced by d-Ala-d-Lac. This is a clinically-significant finding, as this substitution reduces the binding efficacy of the glycopeptide vancomycin by more than 1000-fold. The global rise in infections caused by vancomycin-resistant enterococci (VRE) is a growing concern^27,28^. Therefore, the development of new therapeutics effective against VRE and vancomycin-intermediate/resistant *Staphylococcus aureus* (VISA/VRSA) is urgently needed. Actifensin’s potent activity, demonstrated by its 32-fold greater efficacy against VRE APC1031 compared to plectasin, suggests its potential as a possible candidate for addressing these challenging pathogens. Defensins and other cationic and synthetic antimicrobial peptides have previously garnered some pharmaceutical interest for their therapeutic potential, including NZ2114, a derivative of the fungal defensin plectasin. NZ2114 demonstrated efficacy in vivo^29–31^ but ultimately failed to progress in clinical development. In this study, we highlight the vast, yet largely untapped, diversity of the CSαβ-defensin and defensin-like bacteriocin family across kingdoms, and show beneficial therapeutic characteristics of actifensin, which are similar to non-cytotoxic plectasin^32^ and may be extrapolated across the group of peptides. This emphasises both the substantial opportunities in leveraging this versatile peptide family for clinical application.

Actifensin did not interact and directly depolarise the cell membrane (Fig. 2) as is known for other lipid II-binding peptides like nisin^17^. When delocalisation of MinD was monitored in the presence of actifensin the protein delocalised following 30 min, less than two cell division cycles^33^. Paired with evidence of a weakened cell wall from the visible membrane blebs (Fig. 2a) following actifensin treatment, the killing action of actifensin is most likely due to continuation of the cell division cycle without expansion of the peptidoglycan superstructure, rapidly making the cells susceptible to osmotic pressure and lysis. The structurally-related fungal defensin plectasin was recently demonstrated to oligomerise on bacterial membranes in a calcium-dependent manner^6^. We note the binding of calcium ions was attributed to the negatively-charged patch (Asp-Glu-Asp-Asp) of plectasin, which is absent in actifensin. Oligomerisation activity of plectasin was associated with two histidine side chains (His16 and His18) protruding in opposite orientations from the α-helix, theorised to facilitate inter-peptide interaction. In actifensin only one of these histidines is present, His17 which is highly conserved across CSαβ peptides. Taken together, it remains to be determined if actifensin forms the same supramolecular structures on bacterial membranes, but the presence of additional Ca^2+^ does not affect the inhibitory activity of actifensin indicating that supramolecular assemblies are not formed in the presence of calcium cations like plectasin, and are not part of its mechanism of action. It remains to be determined if such mechanisms are a conserved feature of CSαβ peptides in general, but oligomerisation has been identified for the fungal peptide copsin, which also lacks the residues described for the mechanism in plectasin^6,34^.

Given the conserved nature of residues linked with mechanisms in plectasin, eurocin, and actifensin that are absent in the XTCD group it is likely that the XTCD group may bind to lipid II in a mechanism distinct from that of the GXGCP subfamily which has yet to be determined. Few studies have investigated this group of insect peptides in detail but one peptide, lucifensin produced by green bottle flies (*Lucilia sericata*), complexes with lipid II like actifensin and plectasin^3^. Sapecin, an XTCD-subfamily peptide from flesh flies has been shown to have affinity for cardiolipin^35^, but to our knowledge this has not been tested for lipid II binding activity, and is suggested to form oligomers dependent on an interaction of Asp4 with Arg23^5^, of which Asp4 is highly conserved in the XTCD peptides. This study highlights a gap in the understanding of mechanism-linked structural differences between the two peptide groups, particularly regarding the XTCD residues involved in lipid binding to be further explored to establish the specific mechanism of XTCD peptides.

We also show the conserved nature of bacterial actifensins, which group with the ancient invertebrate type defensins (AITDs) forming the GXGCP CSαβ subfamily. The ancient invertebrate-type defensins are produced by highly diverse eukaryotic taxa including insects, shellfish, and fungi, and have been suggested to share an ancient common eukaryotic or prokaryotic ancestor^36,37^. After our initial discovery of bacterial-encoded defensin structures in gram-positive bacteria, of which most were encoded on similar gene clusters^12^, 74 further defensin-like structures were identified through bioinformatics^38^. Of these, 72 were encoded by species from the phylum Proteobacteria, and one peptide, xanthusin-1 from *Myxococcus xanthus* DK1622, was synthesised and found to exhibit antimicrobial activity. However, its mechanism of action remains uncharacterised^38^. The continued discovery of homologous functional peptides in distantly-related prokaryotes suggests they may have originated from a single common ancestor, but given the immense timescales, size of the peptides and the speed at which bacterial evolution occurs it is difficult to establish a phylogenetic heritage or distinguish between divergent or convergent trajectories of evolution. The defensin superfamily, consisting of cis- and trans-defensins based on the orientation of disulphide bonds, is suggested to have arisen via convergent evolution^2^, and indeed small stable disulphide-rich peptides are an excellent backbone on which to diversify other functions demonstrated by the relatedness of insect and reptile neurotoxins that may have developed from defensin structures^39^. A related toxin structure, the two-disulphide-CSαβ (2ds-CSαβ) fold, present in centipede toxins is widespread in prokaryotic and eukaryotic genomes and has been suggested to predate the split of prokaryotes and eukaryotes^36^. If 2ds-CSαβ peptides represent an ancestor of three-disulphide CSαβ peptides, including actifensin, then the 3ds-CSαβ peptides across eukaryotes and prokaryotes have convergently evolved their structure and lipid II-binding mechanism. However, the 2ds-CSαβ peptide, Sm2, is not antibacterial in nature^36^, indicating that the putative ancestor of 3ds-CSαβ peptides could have been non-antimicrobial peptides that subsequently gained antimicrobial function. Regardless, the remarkable conservation of structure and function of these bacterial peptides with eukaryotic defensins displays a shared evolutionary history of antibacterial weapons, as both a eukaryotic defence mechanism against pathogens or as tools of inter-bacterial competition that are currently under-exploited as human therapeutics.

## Methods

### Heterologous expression of actifensin

Actifensin was heterologously expressed following the protocol outlined by the EasySelect^TM^ Pichia Expression Kit manual. Synthetic DNA encoding the *A. ruminicola* DPC7226 mature AfnA peptide with an N-terminal fusion to α-factor including a kexin cleavage site for mature peptide secretion was synthesised by Genewiz (Germany GmBH, Leipzig). Fastdigest restriction digest, T4 ligase, and Phusion II high-fidelity polymerase enzymes were sourced from Thermo Fisher Scientific (Dublin, Ireland). Inserts were amplified by PCR, before restriction-digestion and ligation with pPICZαA plasmid and subsequent transformation into *E. coli* TOP10 cells (Invitrogen, Dublin, Ireland) plated on low-salt LB agar (1% tryptone, 0.5% yeast extract, 0.5% NaCl) containing 25 µg/ml Zeocin (Invitrogen, Dublin, Ireland). Fragment insertion was confirmed by colony PCR with 5’AOX1 and 3’AOX1 primers prior to plasmid extraction and verification by Sanger sequencing (Genewiz GmBH). Extracted plasmids were linearised, and prepared for transformation into competent *P. pastoris* X-33 by phenol-chloroform purification and ethanol precipitation, prepared per the EasySelect^TM^ Pichia Expression Kit manual. Transformants were plated on YPDS agar (1% yeast extract, 2% peptone, 2% dextrose, 1 M sorbitol, 1.5% agar) containing 100 µg/ml Zeocin and toothpicked onto minimal medium containing methanol or dextrose agar (1.34% yeast nitrogen base, 4 × 10^–5^% biotin, 0.5% methanol or dextrose, and 1.5% agar) and incubated at 30°C aerobically to determine methanol utilisation phenotype. Plates were subsequently overlaid with sloppy MRS agar (0.75% agar) seeded with 0.25% of an overnight culture of *Lactobacillus delbrueckii* ssp. *bulgaricus* LMG6901 and incubated overnight at 37 °C anaerobically. Following inspection for zones of inhibition, the transformant with the largest zone was chosen for expression in liquid culture.

### Actifensin purification

A single colony of *P. pastoris* YIS001 was inoculated into 50 ml buffered minimal glycerol medium in a 250 ml baffled flask and incubated at 30 °C at 225 RPM overnight. The grown culture (OD_600_ > 2.0) was used to seed 500 ml volumes of buffered minimal methanol medium in 2.0 litre baffled flasks which were incubated at 30 °C at 225 RPM supplemented every day with 100% methanol to a final concentration of 0.5% methanol for 7 days. Supernatant was harvested by removal of cells with two rounds of centrifugation at 8000 × *g* for 20 min. The resulting supernatant was subject to actifensin purification as described previously with minor modifications. Briefly, supernatant was applied to 2/3rds of a 30 cm Econo column of XAD-16 beads prewashed with 1 litre of water. The column was washed with 600 ml 25% ethanol and eluted in 70% propan-2-ol containing 0.1% trifluoroacetic acid (IPA-TFA). IPA-TFA was removed from the elute by rotary evaporation, and the sample was diluted 1:1 with deionised water prior to application to a 60 ml C18 column (Phenomenex) pre-equilibrated with methanol and water. The column was washed with 120 ml 20% ethanol and eluted in 100 ml IPA-TFA for further purification with RP-HPLC as described previously^12^. Purity was confirmed with MALDI-TOF mass spectrometry.

### Minimum inhibitory concentrations

MIC values were assayed by microdilution in Mueller-Hinton (MH) broth in low-binding round-bottom polypropylene plates. Selected strains were cultured from glycerol stock on MH agar. For staphylococci, individual colonies were resuspended in 0.9% NaCl to a McFarland of 0.5 and diluted in MH to a final concentration of 5 × 10^5^ CFU/ml in the wells. Other genera were cultured overnight from a single colony, and subcultured to an OD_600_ = 0.5 – 1.0 and diluted to a final concentration of 5 × 10^5^ CFU/ml in the plate. Diluted bacterial suspensions (50 µl) were added to preprepared dilutions of peptide in MH broth (50 µl). Plates were sealed with a gas permeable membrane and incubated for 18–20 h at which point they were visually inspected for growth. The minimum inhibitory concentration was read as the well containing the lowest concentration which completely inhibited growth.

### β-galactosidase stress response bioreporter assay

In brief, reporter strains were cultured in Mueller-Hinton broth containing 5 μg/ml chloramphenicol at 30 °C to an OD_600_ of 0.5. Subsequently, Mueller-Hinton agar was inoculated with 1 × 10^7^ CFU/ml and poured into plates supplemented with 75 μg/ml (cell wall reporter), 125 μg/ml (DNA reporter), and 250 μg/ml (protein and RNA reporters) X-gal, respectively. After the plates were set, 5 μg of actifensin and promoter-inducing control antibiotics were spotted (6 μg vancomycin for cell wall, 0.3 μg ciprofloxacin for DNA, 6 μg rifampicin for RNA, 3 μg clindamycin for protein) and allowed to dry. Results were documented after incubation overnight at 30 °C in the dark.

### Synthesis and purification of peptidoglycan lipid precursors

Large-scale synthesis and purification of peptidoglycan precursors (lipids I and II) were performed as previously described^40^. Pure UDP-N-acetyl-muramic acid pentapeptide (UDP-MurNAc-pp) was produced as described by Kohlrausch and Höltje^41^. Undecaprenyl phosphate (C_55_P) and undecaprenyl diphosphate (C_55_PP) were sourced from Larodan Fine Chemicals AB (Malmö, Sweden) and the phospholipid 1,2-dioleoyl-sn-glycero-3- phosphocholine (DOPC), was purchased from Avanti Polar Lipids (Alabaster, AL, USA). Concentrations of purified peptidoglycan precursor molecules were quantified based on their phosphate content as previously described^42^.

### Precursor complexation assays

Binding of actifensin to peptidoglycan precursors was assayed by incubating 2 nmol of C_55_-P, lipid I, lipid II, and lipid II-d-Lac, and 5 nmol of C_55_-PP with actifensin, at ratios of 1:1, 2:1, and 4:1 (actifensin:precursor) in 30 µl 50 mM Tris/HCl, pH 7.5, for 30 min at room temperature. Complexation was determined by extracting unbound precursors from the mixture using an equal volume of n-butanol/pyridine acetate (pH 4.2) (2:1; vol/vol) analysed by TLC with chloroform/methanol/water/ammonia (88:48:10:1, v/v/v/v) as the solvent and analysed by thin layer chromatography (TLC) with a solvent of chloroform/methanol/water/ammonia (88:48:10:1, v/v/v/v)^43^ that was stained with phosphomolybdic acid^44^. Experiments were performed with biological replicates.

### Isothermal titration calorimetry (ITC)

Large unilamellar vesicles (LUVs) approximately 200 nm in size were prepared from 2 mol% lipid II in DOPC using the extrusion technique^45^. Isothermal titration calorimetry (ITC) experiments were performed at 37 °C using a low-volume Affinity ITC system (TA Instruments). Samples were degassed for 10 min before the experiments. The ITC cell was filled with actifensin (20 mM HEPES, 50 mM NaCl, pH 7.0) and titrated with LUVs in the same buffer under constant stirring at 125 rpm. Data were analysed with Nano Analyze software (version 3.11.0, TA Instruments) by fitting baseline-corrected integrated peaks to an independent binding model. Each measurement was performed in duplicate.

### Induction and antagonization of *lia-lux* cell wall stress response

*B. subtilis* P*lia-lux*^46^ was cultured from an overnight preculture in MH broth containing chloramphenicol (5 μg/ml) to an OD_600_ of 0.6. Actifensin and vancomycin (Hikma Pharma GmbH) were added to a Greiner LUMITRAC^TM^ 96-well-microtiter plate at serially diluted concentrations before addition of an equal volume of the reporter strain for final concentrations ranging from 64 to 0.0625 µg/ml. Cell wall stress was measured as an increase in luminescence with a Tecan Infinite M200 microplate reader for 10 h at 30 °C.

Antagonization of the actifensin-induced *lia-lux* response was used to corroborate the complex formation between actifensin and peptidoglycan precursors, as described previously^47^. Purified C_55_-P, C_55_-PP, lipid I, and lipid II, were preincubated with actifensin at molar ratios of 1:1, 2:1, and 4:1 (precursor:actifensin) for 15 min at room temperature. After preincubation, the reporter strain was added and luminescence was measured as described above. Measurements were performed with at least three independent biological replicates.

### DiSC_3_(5) efflux assay

To measure efflux of membrane-associated DiSC_3_(5), *B. subtilis* 168 was subcultured from an overnight culture and grown to an OD_600_ of 0.3. DiSC_3_(5) (Biomol, Germany) was added to a final concentration of 1 μM. A total of 200 μl of the culture was added to a Greiner Bio-One flat-bottom black polystyrene 96-well microtiter plate. Fluorescence was measured using a Tecan Infinite 200 Pro microplate reader equipped with a monochromator with 580-nm excitation and 635-nm emission wavelengths every minute for 10 min until the signal remained stable, indicating maximal dye-uptake and therefore auto-quenching of fluorescence. Actifensin was added at 1x, 3x, and 6x fold MIC and DiSC_3_(5) fluorescence was measured for 60 min. Valinomycin at a concentration of 1 µM was used as a positive control for membrane depolarisation. Measurements were performed with at least three independent biological replicates.

### Bacterial cell wall integrity assay

Bacterial cell wall integrity assays were performed as described previously with minor modifications^48^. *B. subtilis* 168 was subcultured from an overnight culture in MH broth at 30 °C to an OD_600_ of 0.3. Log phase cells were then separated and treated with actifensin at 0.5 and 1x fold MIC, 2 µg/ml vancomycin, 0.5 µg/ml nisin, 2 µg/ml bacitracin, and 128 µg/ml ciprofloxacin, and further incubated for 90 min at 30 °C. A total of 200 µl volumes of cells were then fixed by addition of 800 µl 1:3 (v:v) mixture of acetic acid and methanol, and immobilised on thin 1% w/v agarose containing 0.9% NaCl slides. Imaging was performed by phase contrast microscopy on a Carl Zeiss Axio Observer Z1 equipped with a Colibri 5/7 LED, a Carl Zeiss Plan-Apochromat 100x/1.40 Oil Ph 3 M27 objective, and a Carl Zeiss Axiocam 820 mono camera. Visualisation was achieved with an exposure time of 1 ms. Five to ten images of different fields of view were acquired under each condition using the Carl Zeiss Zen Blue 2.0 software and postprocessing was performed using ImageJ2 v2.14.0 software^49^. Cells displaying bleb formation were counted manually and expressed as a percentage of total cells.

### MinD assay

*B. subtilis 168 ameE::spc P*_*xyl*_*-gfp-minD*, encoding GFP-tagged *minD* under control of the P*xyl* promotor^18^, was grown in LB broth supplemented with 0.1% w/v xylose, 0.2% dextrose to prevent sporulation, and 50 µg/ml spectinomycin overnight at 30 °C, and subcultured without spectinomycin or dextrose to an OD_600_ of 0.3. Imaging was performed within 5 min after addition of actifensin at 6x MIC. Nisin at 13 µg/ml was used as positive control. Samples were immobilised on microscope slides covered with 1% w/v agarose. Widefield fluorescence microscopy was performed on a Carl Zeiss AxioObserver Z1 equipped with a Colibri 5/7 LED, a Carl Zeiss Plan-Apochromat 100x/1.40 Oil Ph 3 M27 objective, and a Carl Zeiss Axiocam 820 mono camera. Optical sectioning was performed with the Apotome 3. Visualisation was achieved with an exposure time of 350 ms and the Carl Zeiss filters 501-527 (excitation wavelength 450–488 nm, emission wavelength 501–527 nm). The setup was controlled using the Carl Zeiss Zen Blue 2.0 software. A minimum of five separate images were acquired at 5 min, 15 min, 30 min, and 60 min post-peptide addition with ZEN 2 software (Zeiss) and analysis and postprocessing were performed using ImageJ2 v2.14.0^49^.

### Liposome destruction assay

Large unilamellar vesicles containing carboxyfluorescein were prepared from DOPC and DOPC containing 0.5 mol% lipid II. Two micromoles DOPC or 1990 nmol with 10 nmol lipid II were desiccated in round bottom glass tubes and resuspended by vigorous vortexing in TBS (10 mM Tris/HCl, pH 7.2, 0.85% w/v NaCl) buffer containing 50 mM carboxyfluorescein (CF) dissolved in 1 M NaOH. The resuspended lipid mixture was subjected to 10 rounds of freezing in liquid nitrogen and thawing in a 30 °C water bath before extrusion through a 4-µm pore filter 10 times using an Avanti Mini-extruder (Avanti Polar Lipids, Alabaster, USA). Extruded lipid solutions were run on a Sephadex G-50 column to remove free CF, and the liposome-containing-eluent was assayed for phosphate concentration, to measure liposome concentration, as described previously. Liposome destruction was determined by measuring fluorescence from CF release following addition of peptides with the addition of Triton X-100 to test maximum release. Prepared liposomes were used on the same day as preparation.

All measurements were conducted on a Shimadzu HYPER-RF fluorometer (Shimadzu). Liposome solutions were diluted to a 25 µM lipid Pi concentration in TBS and added to a 2 ml quartz cuvette stirred magnetically. The solution was excited at 492 nm, measuring emission at 520 nm with intervals of 1 s for 3 min, with a 30 s equilibration before peptide addition, 2 min measurement followed by addition of 20% Triton-X 100 for maximum lysis.

### Biofilm inhibition

*S. aureus* ATCC25923 was cultured in TSB with 0.5% glucose at 37 °C until OD_600_ reached 0.5–1. Antibiotic dilutions were prepared in MBEC Biofilm Inoculator 96-well plates (Innovotech, Edmonton, Canada) using 100 µl TSB with 0.5% glucose, 2% DMSO, and, for dalbavancin 0.004% Tween80. A bacterial suspension was diluted to 10^6^ CFU/ml, and 100 µl was added to each well. Plates were incubated overnight at 37 °C with shaking (60 rpm) in a humidified incubator, and initial inoculum density was verified by colony counting. Following incubation, plates were rinsed with 0.9% NaCl, and the peg lid was transferred to a new plate containing TSB with 0.5% glucose. Biofilms were detached by sonication for 30 min at maximum settings. CFUs from biofilm growth controls were determined. The recovery plate was incubated for 24 h at 37 °C, and the minimum inhibitory concentration (MIC) was assessed by an additional 24-h incubation of the challenge plate. Minimum bactericidal concentration (MBC) was determined by transferring 20 µl from the challenge plate to a new TSB plate, followed by 24-h incubation. Residual biofilms were quantified by measuring absorbance of the recovery plate at 650 nm using a plate reader.

### Mammalian cytotoxicity

Cytotoxicity of actifensin on human epithelial type G2 (Hep G2) cells (ATCC HB-8065) was measured by using the non-fluorescent resazurin-based alamarBlue^TM^ cell viability reagent (Invitrogen) which is converted by living cells into fluorescent resorufin. Hep G2 cells were seeded at a density of 3.5 × 10^4^ cells per well in 96-well flat base TC plates (Sarstedt), and incubated in Dulbecco’s modified Eagle’s medium (DMEM, Gibco) supplemented with 1× MEM non-essential amino acids (Gibco) and 1× MEM vitamin solution (Gibco) in an atmosphere of 5% CO_2_ at 37 °C. After 72 h, the culture was treated with actifensin at serially diluted concentrations ranging from 1 to 128 μg/ml. After incubation for 24 h, the medium was removed and the cell monolayer was washed twice with Hank’s balanced salt solution (HBSS, Gibco). To indicate cell viability, alamarBlueTM reagent was added to a final concentration of 10% (v/v) and cells were incubated for 1 h at 37 °C and 5% CO_2_. Fluorescence measurements were performed in black F-bottom microplates (FLUOTRAC, Greiner) with a microplate reader Spark 10 M (Tecan) at 570 nm excitation and 585 nm emission. Relative cell viability was calculated as the percentage of untreated cells (set to 100%).

### Red blood cell lysis assay

Human red blood cells (RBCs) used in this assay were obtained from the University Clinic Bonn as erythrocyte concentrate. One ml concentrated RBCs were diluted 1:10 in PBS pH 7.2 (Gibco) and centrifuged at 2000 × *g* for 10 min. The supernatant was discarded, and the RBCs were resuspended in PBS to a final OD_600_ of 24. In a 96-well flat base TC plate (Sarstedt) actifensin was serially diluted in PBS with concentrations ranging from 1 to 128 µg/ml. RBCs were added in an equal volume and incubated at 37 °C and 5% CO_2_. After 24 h, RBCs were pelleted by centrifugation (1500 × *g* for 10 min) and the supernatants were diluted 5-fold in PBS in a new 96-well plate. Absorbance of the heme was measured at 405 nm in a GloMax Explorer (Promega). Relative haemolysis was calculated as the percentage of RBCs treated with 1% (v/v) Triton X-100 (set 100%).

### Immunomodulation in PBMCs

PBMCs were isolated from buffy coat donations provided by the Institute of Experimental Haematology and Transfusion Medicine, University Hospital Bonn. The studies with human blood were approved by the ethics committee of the University Clinic Bonn (315/22) and written informed consent was obtained from all healthy donors. Buffy coat samples were processed using density gradient centrifugation with Biocoll separation media (BS L6115, Bio&SELL, Nuremberg, Germany). PBMCs were seeded in 24-well plates at a density of 5 × 10^6^ cells per well in serum-free RPMI 1640 medium supplemented with L-glutamine (21875034, Life Technologies, Darmstadt, Germany). PBMCs were pre-stimulated with either actifensin (10 µM) or LL-37 (10 µM; tlrl-l37-5, InvivoGen, Toulouse, France) for 30 min. Afterwards, cells were stimulated with Pam_3_CSK_4_ (tlrl-pms-1, InvivoGen, Toulouse, France), LPS (tlrl-3pelps, InvivoGen, Toulouse, France), or *S. aureus* RN4220 for 3 h. Cell-free culture supernatants were collected and analysed for cytokine release using a TNF ELISA kit (88-7346-88, Thermo Fisher Scientific, Darmstadt, Germany). For LDH release analysis, PBMCs were incubated with varying concentrations of actifensin for 3.5 h. The culture supernatants were collected and analysed using an LDH Cytotoxicity Detection Kit (C20300, Invitrogen, Toulouse, France).

### In silico structural comparative analysis

To compare mechanism-related structural components of actifensin and related bacterial and eukaryotic peptides, reviewed members of the arthropod defensin protein family (PFAM accession PF01097) and hidden Markov model identified actifensins (NCBI accession NF038042.1) were downloaded and trimmed to mature peptide sequences based on homology and PFAM predicted leader sequences. The mature peptide sequences were aligned using MUSCLE (version 3.8.425) with default parameters and subsequently manually adjusted to align conserved cysteine residues. Where protein data bank structures were available structures were visualised and aligned to the X-ray crystallography model of plectasin, using PyMOL (version 3.0.0) and where unavailable, 3-D models were generated using AlphaFold3. Residues of known function in Plectasin were highlighted and their presence or substitution was compared with other peptides.

### Defensin and bacteriocin taxonomic mapping

Taxonomic ID accessions were extracted from PFAM and NF accessions (Table) and entered into the Lifemap Tree of Life web platform containing the entire NCBI taxonomic entries (updated Oct 2023)^50^. A tree of taxa encoding arthropod defensin or actifensin peptides was downloaded and visualised using iToL^51^ (v6), which was subsequently exported and overlaid with NCBI common taxonomy names.

### Statistical analysis

Bar graphs show mean + SEM. For single comparisons, significant differences were determined using a two-tailed Student’s *t* test. One-way ANOVA followed by Dunnett’s post test was used to analyse statistically significant differences for multiple comparisons. Differences were considered statistically significant at *p* ≤ 0.05. All statistical analyses were performed with GraphPad Prism software version 10.3.

## Supplementary information

supplementary_data.

## Data Availability

The raw data supporting the findings of this study are available from the corresponding author upon reasonable request.

## References

[1] Sugrue, I., Ross, R. P. & Hill, C. Bacteriocin diversity, function, discovery and application as antimicrobials. *Nat. Rev. Microbiol.*10.1038/s41579-024-01045-x (2024). 10.1038/s41579-024-01045-x PMC7616364 38730101

[2] Shafee, T. M., Lay, F. T., Hulett, M. D. & Anderson, M. A. The defensins consist of two independent, convergent protein superfamilies. *Mol. Biol. Evol.* **33**, 2345–2356 (2016). 27297472 10.1093/molbev/msw106

[3] Schneider, T. et al. Plectasin, a fungal defensin, targets the bacterial cell wall precursor Lipid II. *Science* **328**, 1168–1172 (2010). 20508130 10.1126/science.1185723

[4] Gueguen, Y. et al. Characterization of a defensin from the oyster Crassostrea gigas. Recombinant production, folding, solution structure, antimicrobial activities, and gene expression. *J. Biol. Chem.* **281**, 313–323 (2006). 16246846 10.1074/jbc.M510850200

[5] Takeuchi, K. et al. Channel-forming membrane permeabilization by an antibacterial protein, sapecin. *J. Biol. Chem.* **279**, 4981–4987 (2004). 14630928 10.1074/jbc.M307815200

[6] Jekhmane, S. et al. Host defence peptide plectasin targets bacterial cell wall precursor lipid II by a calcium-sensitive supramolecular mechanism. *Nat. Microbiol.*10.1038/s41564-024-01696-9 (2024). 10.1038/s41564-024-01696-9 PMC11222147 38783023

[7] Sass, V. et al. Human beta-defensin 3 inhibits cell wall biosynthesis in Staphylococci. *Infect. Immun.* **78**, 2793–2800 (2010). 20385753 10.1128/IAI.00688-09 PMC2876548

[8] Oeemig, J. S. et al. Eurocin, a new fungal defensin: structure, lipid binding, and its mode of action. *J. Biol. Chem.* **287**, 42361–42372 (2012). 23093408 10.1074/jbc.M112.382028 PMC3516779

[9] Field, D., Fernandez de Ullivarri, M., Ross, R. P. & Hill, C. After a century of nisin research—where are we now?. *FEMS Microbiol. Rev.* **47**, fuad023 (2023). 37300874 10.1093/femsre/fuad023 PMC10257480

[10] Zhu, L., Zeng, J. & Wang, J. Structural basis of the immunity mechanisms of pediocin-like bacteriocins. *Appl. Environ. Microbiol.* **88**, e00481–22 (2022). 35703550 10.1128/aem.00481-22 PMC9275228

[11] Imai, Y. et al. A new antibiotic selectively kills Gram-negative pathogens. *Nature* **576**, 459–464 (2019). 31747680 10.1038/s41586-019-1791-1 PMC7188312

[12] Sugrue, I., O’Connor, P. M., Hill, C., Stanton, C. & Ross, R. P. Actinomyces produces defensin-like bacteriocins (actifensins) with a highly degenerate structure and broad antimicrobial activity. *J. Bacteriol.* **202**, e00529-19 (2020). 10.1128/JB.00529-19 PMC6989792 31767775

[13] Harms, H. et al. Antimicrobial dialkylresorcins from marine-derived microorganisms: insights into their mode of action and putative ecological relevance. *Planta Med.* **84**, 1363–1371 (2018). 29991081 10.1055/a-0653-7451

[14] Reithuber, E. et al. THCz: small molecules with antimicrobial activity that block cell wall lipid intermediates. *Proc. Natl. Acad. Sci. USA* **118**, e2108244118 (2021). 34785593 10.1073/pnas.2108244118 PMC8617507

[15] Shukla, R. et al. An antibiotic from an uncultured bacterium binds to an immutable target. *Cell* **186**, 4059–4073.e27 (2023). 37611581 10.1016/j.cell.2023.07.038

[16] Mascher, T., Zimmer, S. L., Smith, T.-A. & Helmann, J. D. Antibiotic-inducible promoter regulated by the cell envelope stress-sensing two-component system LiaRS of *Bacillus subtilis*. *Antimicrob. Agents Chemother.* **48**, 2888–2896 (2004). 15273097 10.1128/AAC.48.8.2888-2896.2004 PMC478541

[17] Hasper, H. E., De Kruijff, B. & Breukink, E. Assembly and stability of nisin−Lipid II pores. *Biochemistry* **43**, 11567–11575 (2004). 15350143 10.1021/bi049476b

[18] Strahl, H. & Hamoen, L. W. Membrane potential is important for bacterial cell division. *Proc. Natl. Acad. Sci. USA* **107**, 12281–12286 (2010). 20566861 10.1073/pnas.1005485107 PMC2901462

[19] Te Winkel, J. D., Gray, D. A., Seistrup, K. H., Hamoen, L. W. & Strahl, H. Analysis of antimicrobial-triggered membrane depolarization using voltage sensitive dyes. *Front. Cell Dev. Biol.* **4**, 29 (2016). 27148531 10.3389/fcell.2016.00029 PMC4829611

[20] Mookherjee, N., Anderson, M. A., Haagsman, H. P. & Davidson, D. J. Antimicrobial host defence peptides: functions and clinical potential. *Nat. Rev. Drug Discov.* **19**, 311–332 (2020). 32107480 10.1038/s41573-019-0058-8

[21] Hancock, R. E. & Sahl, H.-G. Antimicrobial and host-defense peptides as new anti-infective therapeutic strategies. *Nat. Biotechnol.* **24**, 1551–1557 (2006). 17160061 10.1038/nbt1267

[22] Van Dalen, R., Peschel, A. & Van Sorge, N. M. Wall teichoic acid in Staphylococcus aureus host interaction. *Trends Microbiol.* **28**, 985–998 (2020). 32540314 10.1016/j.tim.2020.05.017

[23] Wanner, S. et al. Wall teichoic acids mediate increased virulence in Staphylococcus aureus. *Nat. Microbiol.* **2**, 16257 (2017). 28112716 10.1038/nmicrobiol.2016.257

[24] Dimarcq, J. L., Bulet, P., Hetru, C. & Hoffmann, J. Cysteine-rich antimicrobial peptides in invertebrates. *Biopolymers* **47**, 465–477 (1998). 10333738 10.1002/(SICI)1097-0282(1998)47:6<465::AID-BIP5>3.0.CO;2-#

[25] Oppedijk, S. F., Martin, N. I. & Breukink, E. Hit ’em where it hurts: the growing and structurally diverse family of peptides that target lipid-II. *Biochim. Biophys. Acta* **1858**, 947–957 (2016). 26523408 10.1016/j.bbamem.2015.10.024

[26] Zhu, S. Adaptively evolved human oral actinomyces-sourced defensins show therapeutic potential. *EMBO Mol. Med.* **14**, e14499 (2021). 34927385 10.15252/emmm.202114499 PMC8819291

[27] Cimen, C. et al. Vancomycin-resistant enterococci (VRE) in hospital settings across European borders: a scoping review comparing the epidemiology in the Netherlands and Germany. *Antimicrob. Resist. Infect. Control* **12**, 78 (2023). 37568229 10.1186/s13756-023-01278-0 PMC10422769

[28] Thomsen, J. et al. Epidemiology of vancomycin-resistant enterococci in the United Arab Emirates: a retrospective analysis of 12 years of national AMR surveillance data. *Front. Public Health* **11**, 1275778 (2023). 38089023 10.3389/fpubh.2023.1275778 PMC10715431

[29] Xiong, Y. Q. et al. Efficacy of NZ2114, a novel plectasin-derived cationic antimicrobial peptide antibiotic, in experimental endocarditis due to methicillin-resistant Staphylococcus aureus. *Antimicrob. Agents Chemother.* **55**, 5325–5330 (2011). 21859940 10.1128/AAC.00453-11 PMC3195053

[30] Andes, D., Craig, W., Nielsen, L. A. & Kristensen, H. H. In vivo pharmacodynamic characterization of a novel plectasin antibiotic, NZ2114, in a murine infection model. *Antimicrob. Agents Chemother.* **53**, 3003–3009 (2009). 19414576 10.1128/AAC.01584-08 PMC2704636

[31] Østergaard, C., Sandvang, D., Frimodt-Møller, N. & Kristensen, H.-H. High cerebrospinal fluid (CSF) penetration and potent bactericidal activity in CSF of NZ2114, a novel plectasin variant, during experimental pneumococcal meningitis. *Antimicrob. Agents Chemother.* **53**, 1581–1585 (2009). 19188395 10.1128/AAC.01202-08 PMC2663087

[32] Hara, S. et al. Plectasin has antibacterial activity and no affect on cell viability or IL-8 production. *Biochem. Biophys. Res. Commun.* **374**, 709–713 (2008). 18675251 10.1016/j.bbrc.2008.07.093

[33] Guo, T. & Herman, J. K. Magnesium modulates bacillus subtilis cell division frequency. *J. Bacteriol.* **205**, e00375–22 (2023). 36515540 10.1128/jb.00375-22 PMC9879117

[34] Essig, A. et al. Copsin, a novel peptide-based fungal antibiotic interfering with the peptidoglycan synthesis. *J. Biol. Chem.* **289**, 34953–34964 (2014). 25342741 10.1074/jbc.M114.599878 PMC4263892

[35] Matsuyama, K. & Natori, S. Mode of action of sapecin, a novel antibacterial protein of Sarcophaga peregrina (flesh fly). *J. Biochem.* **108**, 128–132 (1990). 2172219 10.1093/oxfordjournals.jbchem.a123151

[36] Dash, T. S. et al. A centipede toxin family defines an ancient class of CSαβ defensins. *Structure* **27**, 315–326.e7 (2019). 30554841 10.1016/j.str.2018.10.022

[37] Zhu, S. Evidence for myxobacterial origin of eukaryotic defensins. *Immunogenetics* **59**, 949–954 (2007). 18058146 10.1007/s00251-007-0259-x

[38] Cardoso, M. H. et al. Discovery of five classes of bacterial defensins: ancestral precursors of defensins from eukarya?. *ACS Omega* **9**, 45297–45308 (2024). 39554447 10.1021/acsomega.4c06956 PMC11561630

[39] Meng, L. et al. Scorpion potassium channel-blocking defensin highlights a functional link with neurotoxin. *J. Biol. Chem.* **291**, 7097–7106 (2016). 26817841 10.1074/jbc.M115.680611 PMC4807291

[40] Ling, L. L. et al. A new antibiotic kills pathogens without detectable resistance. *Nature* **517**, 455–459 (2015). 25561178 10.1038/nature14098 PMC7414797

[41] Kohlrausch, U. & Höltje, J. V. Analysis of murein and murein precursors during antibiotic-induced lysis of Escherichia coli. *J. Bacteriol.* **173**, 3425–3431 (1991). 2045364 10.1128/jb.173.11.3425-3431.1991 PMC207955

[42] Rouser, G., Fleischer, S. & Yamamoto, A. Two dimensional thin layer chromatographic separation of polar lipids and determination of phospholipids by phosphorus analysis of spots. *Lipids* **5**, 494–496 (1970). 5483450 10.1007/BF02531316

[43] Rick, P. D. et al. Characterization of the lipid-carrier involved in the synthesis of enterobacterial common antigen (ECA) and identification of a novel phosphoglyceride in a mutant of Salmonella typhimurium defective in ECA synthesis. *Glycobiology* **8**, 557–567 (1998). 9592122 10.1093/glycob/8.6.557

[44] Schneider, T. et al. In vitro assembly of a complete, pentaglycine interpeptide bridge containing cell wall precursor (lipid II-Gly _5_) of *Staphylococcus aureus*. *Mol. Microbiol.* **53**, 675–685 (2004). 15228543 10.1111/j.1365-2958.2004.04149.x

[45] Hope, M. J., Bally, M. B., Webb, G. & Cullis, P. R. Production of large unilamellar vesicles by a rapid extrusion procedure. Characterization of size distribution, trapped volume and ability to maintain a membrane potential. *Biochim. Biophys. Acta Biomembr.* **812**, 55–65 (1985). 10.1016/0005-2736(85)90521-8 23008845

[46] Radeck, J. et al. Anatomy of the bacitracin resistance network in *B**acillus subtilis*. *Mol. Microbiol.* **100**, 607–620 (2016). 26815905 10.1111/mmi.13336

[47] Deisinger, J. P. et al. Dual targeting of the class V lanthipeptide antibiotic cacaoidin. *iScience* **26**, 106394 (2023). 37013189 10.1016/j.isci.2023.106394 PMC10066520

[48] Wenzel, M. et al. Proteomic response of Bacillus subtilis to lantibiotics reflects differences in interaction with the cytoplasmic membrane. *Antimicrob. Agents Chemother.* **56**, 5749–5757 (2012). 22926563 10.1128/AAC.01380-12 PMC3486579

[49] Schindelin, J. et al. Fiji: an open-source platform for biological-image analysis. *Nat. Methods* **9**, 676–682 (2012). 22743772 10.1038/nmeth.2019 PMC3855844

[50] De Vienne, D. M. Lifemap: exploring the entire tree of life. *PLoS Biol.* **14**, e2001624 (2016). 28005907 10.1371/journal.pbio.2001624 PMC5179005

[51] Letunic, I. & Bork, P. Interactive Tree of Life (iTOL) v6: recent updates to the phylogenetic tree display and annotation tool. *Nucleic Acids Res.* **52**, W78–W82 (2024). 38613393 10.1093/nar/gkae268 PMC11223838

