# Supplementary material for: Trans-kingdom conservation of mechanism between bacterial actifensin and eukaryotic defensins
Source: NPJ Antimicrob Resist. 2025 Jul 22;3:66. doi: 10.1038/s44259-025-00135-x (PMC12284004; doi:10.1038/s44259-025-00135-x)
Supplement: Supplementary file 1 — supplementary_data. [file 44259_2025_135_MOESM1_ESM.docx]

**Supplementary Table 1.** Minimum inhibitory concentrations (MICs) of actifensin in the presence and absence of calcium (1.25 mM).

| *Species* | Strain | Actifensin MIC  µg/ml (µM) | |
| --- | --- | --- | --- |
|  |  | 0 mM Cacl | 1.25 mM Cacl |
| *Micrococcus luteus* |  | 0.25 (0.06) | 0.25 (0.06) |
| *Staphylococcus aureus* | HG001 | 4.0 (0.98) | 4.0 (0.98) |
| *Staphylococcus aureus* | HG001 DapR | 4.0 (0.98) | 4.0 (0.98) |
| *Staphylococcus aureus* | SA113 | 4.0 (0.98) | 4.0 (0.98) |
| *Staphylococcus aureus* | SA113 ∆tarO | 4.0 (0.98) | 4.0 (0.98) |
| *Staphylococcus aureus* | SA113 ∆LTA | 4.0 (0.98) | 4.0 (0.98) |
| *Staphylococcus aureus* | SA113 ∆dlt | 1.0 (0.24) | 1.0 (0.24) |

**Supplementary Table 2.** Summary table of thermodynamic parameters generated by the titrations of LI/DOPC and LII/DOPC LUVs into 20 µM actifensin solutions in buffer (20 mM HEPES, 50 mM NaCl, pH 7.0) at 25 °C.

| **N** | **Kd (nM)** | **∆H (kJ/mol)** | **∆G (kJ/mol)** | **-T∆S (kJ/mol)** |
| --- | --- | --- | --- | --- |
| 0.800 | 10.7 | -27.0 | -45.5 | -18.6 |
| 0.888 | 6.5 | -26.0 | -46.8 | -20.7 |
| 0.795 | 55.8 | -28.5 | -41.4 | -13.0 |
|  |  |  |  |  |
| 0.831 | 50.4 | -33.5 | -41.7 | -8.2 |
| 0.793 | 15.6 | -35.3 | -44.6 | -9.3 |
| 0.827 | 27.1 | -35.0 | -43.2 | -8.2 |

**Supplementary Figure 1.** Thin-layer chromatography membranes showing that actifensin (Afn) complexes with lipids I (LI), II (LII), and lipid II-D-lac (LII-D-Lac), but not with C_55_-P or C_55_-PP. Controls include bacitracin (Baci) and vancomycin (Vanc) for C_55_-PP, LI and LII assays respectively.


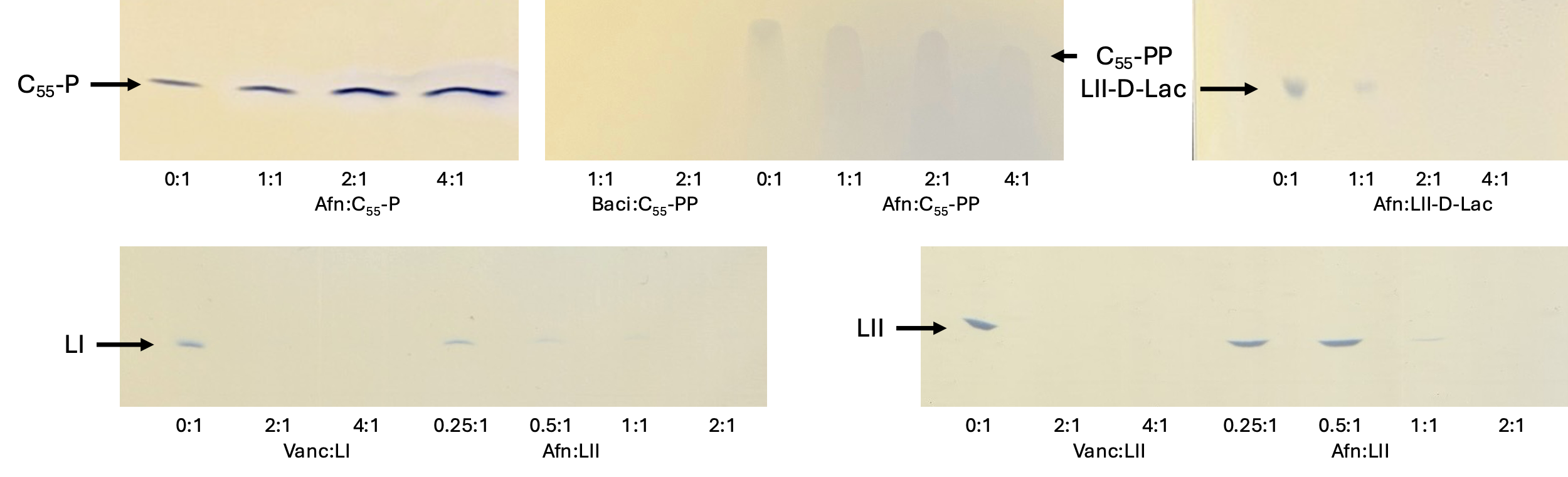


**Supplementary Figure 2. Actifensin interferes induces cell wall stress.** Effect of actifensin on *B. subtilis* P_lia_-lux, which expresses luciferase under the control of the *lia* cell wall stress response promoter.


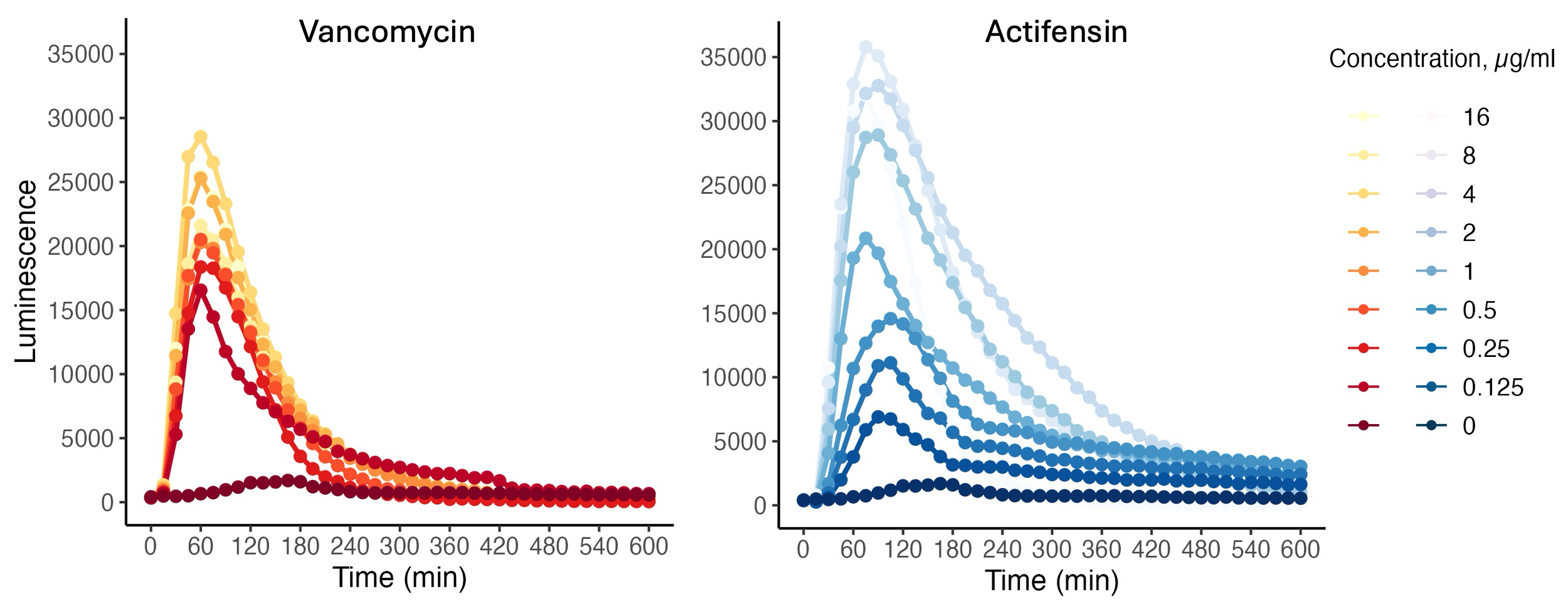


**Supplementary Figure 3. Actifensin complexes with cell wall precursors.** Detailed analysis from Fig. 1c: Impact of increasing lipid-to-actifensin ratios (1:1, 2:1, 4:1) on the actifensin-induced *lia* cell wall stress response.

**Supplementary Figure 4.** Isothermal titration calorimetry binding thermograms of (a) DOPC/Lipid I, (b) DOPC/Lipid II, and (c) control titrations of blank DOPC large unilamellar vesicles into actifensin. Raw data (top, differential power in µW) and integrated data (bottom, kJ/mol) diagrams for LI/LII (200 µM) containing DOPC LUVs (10 mM) titrated into a solution of ACT (20 µM) in 20 mM HEPES, 50 mM NaCl, pH 7.0 at 25 °C.


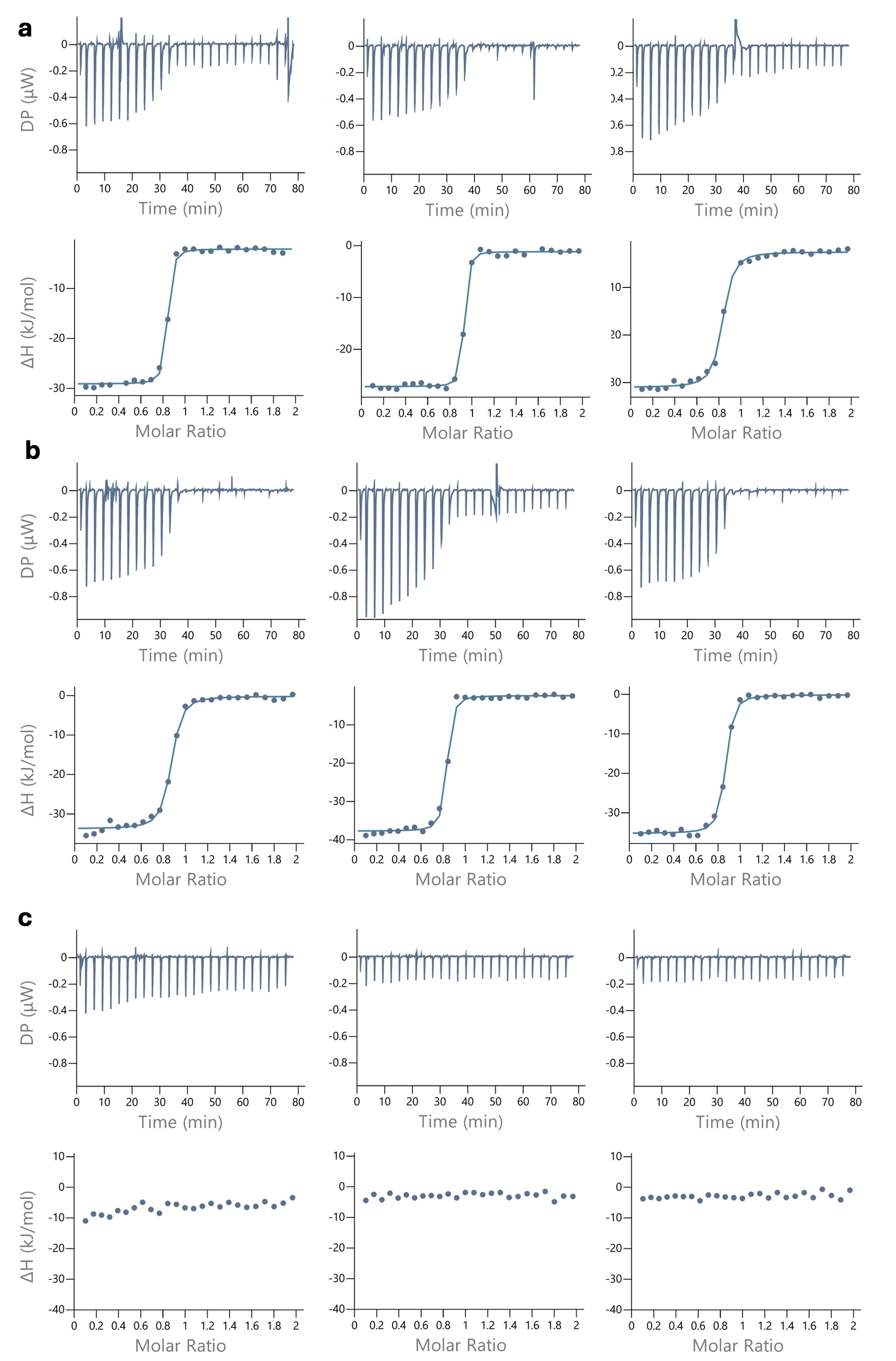


**Supplementary Figure 5. Comparison of actifensin (AfnA) and the potent biofilm inhibitor dalbavancin (Dalba) in inhibition of biofilm formation by *S. aureus* ATCC25923.**

**Supplementary Figure 6**. Effect of actifensin on LDH release in PBMCs. Scatter dot plots show mean as bars.

**Supplementary Figure 7.** Sequence alignment of actifensins (sequences beginning with WP_) with CS⍺β eukaryotic defensins, selected named peptides highlighted with arrows. Top: cartoon overlay demonstrating the secondary structure of CS⍺β defensins and disulphide bond pattern.

**
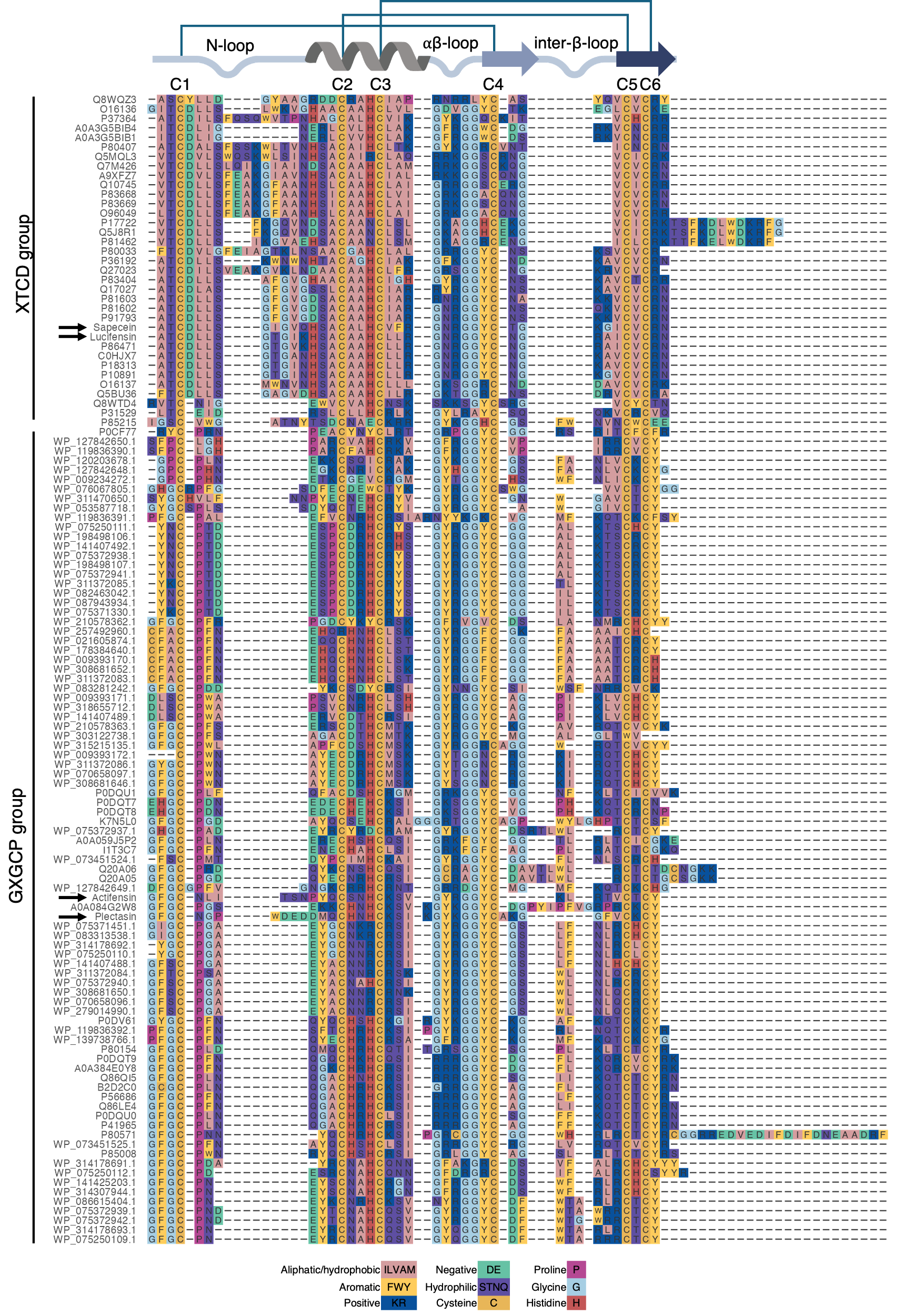
**
